# Hematopoietic Stem Cells Transplant (HSCT)-Related Chronic Pulmonary Diseases: An Overview

**DOI:** 10.3390/children10091535

**Published:** 2023-09-11

**Authors:** Arianna Traunero, Francesca Peri, Laura Badina, Alessandro Amaddeo, Elettra Zuliani, Massimo Maschio, Egidio Barbi, Sergio Ghirardo

**Affiliations:** 1 Department of Medical, Surgical and Health Sciences, University of Trieste, 34126 Trieste, Italy; 2 Department of Pediatrics, Institute for Maternal and Child Health, IRCCS Burlo Garofolo, 34137 Trieste, Italy; 3 Emergency Department, Institute for Maternal and Child Health, IRCCS Burlo Garofolo, 34137 Trieste, Italy

**Keywords:** pulmonary graft versus host disease, hematopoietic stem cells transplantation, late-onset non-infectious pulmonary complications, chronic graft versus host disease

## Abstract

Recipients of HSCT have a high risk of infective and non-infective pulmonary diseases. Most patients with pulmonary involvement present multiple pathogenetic mechanisms simultaneously with complex interactions. Therefore, it can be difficult to distinguish the contributions of each one and to perform studies on this subject. In this opinion article, we discuss only chronic pulmonary manifestations, focusing on LONIPCs (late-onset non-infectious pulmonary complications). This term embraces drug-related toxicity, allergies, and chronic pulmonary graft versus host disease (GvHD) in all its recently identified clinical variants. Among LONIPCs, GvHD represents the most critical in terms of morbidity and mortality, despite the rapid development of new treatment options. A recently emerging perspective suggests that pulmonary lung rejection in transplant patients shares striking similarities with the pathogenesis of GvHD. In a pulmonary transplant, the donor organ is damaged by the host immune system, whereas in GvHD, the donor immune system damages the host organs. It constitutes the most significant breakthrough in recent years and is highly promising for both hematologists and thoracic transplant surgeons. The number of patients with LONIPCs is scarce, with heterogenous clinical characteristics often involving several pathogenetic mechanisms, making it challenging to conduct randomized controlled trials. Therefore, the body of evidence in this field is scarce and generally of low quality, leading to jeopardized choices in terms of immunosuppressive treatment. Moreover, it risks being outdated by common practice due to the quick evolution of knowledge about the diagnosis and treatment of LONIPCs. The literature is even more pitiful for children with pulmonary involvement related to HSCT.

## 1. Introduction

This opinion article aims to give a narrative review of the several pathogenetic mechanisms that contribute to and overlap in the development of hematopoietic stem cell transplantation (HSCT)-related chronic pulmonary diseases in children.

Pulmonary management of patients who have undergone HSCT represents a formidable challenge due to the scarce and sparse evidence about the various HSCT-related conditions. Such difficulty in obtaining good-quality evidence is even more remarkable regarding children’s literature. Nevertheless, chronic pulmonary complications after HSCT affect a significant percentage of patients. When considering late-onset non-infectious pulmonary complications (LONIPCs) exclusively, they affect around one-fifth of HSCT recipients, representing the major long-term cause of morbidity and mortality after the first 4 years post-HSCT. Early recognition and aggressive management are crucial to improving patient outcomes.

When approaching pulmonary disease after HSCT, it is essential to delve into the complex interactions of several pathogenetic mechanisms that concur. The underlying disease leading to HSCT may play a significant role after the procedure, either directly or more often indirectly: tissue damage secondary to the treatments received (chemo-/radiotherapy); opportunistic or chronic infections may all contribute to lung injury pathogenesis; pulmonary graft versus host disease (pGvHD) often has a strong impact on the patient’s prognosis. Additionally, donor allergies can be acquired through the HSCT. Recently, the concept that pGvHD and lung rejection share many more similar aspects than differences has gained traction, with a broad body of knowledge and different viewpoints suddenly becoming available [1].

Most patients with pulmonary involvement secondary to HSCT usually present a clinical picture characterized by the entangled interaction of these conditions. Therefore, it is challenging to study every condition and their interaction separately. An effort is needed to understand all these possible pathogenetic mechanisms better. This review aims to provide a checklist of the most frequent mechanisms that may help optimize treatment timing and management.

## 2. Underling Disease before HSCT

In childhood, HSCT is the treatment of choice for several conditions, mostly life-threatening hematologic malignancies and congenital immune deficiencies or diseases. HSCT can significantly improve patients’ outcomes, and, in some cases, it leads to complete healing. However, HSCT may involve systemic complications that can worsen a previous tissue or organ dysfunction related to the underlying disease. Regarding hematologic malignancies, pulmonary complications may be related to pneumonia, hemorrhage, oedema, and chemotherapy-related toxicity due to drugs received before the HSCT. Lung disease can result from leukemic pulmonary infiltration, which may be diffuse or focal, affecting the parenchyma, alveoli, bronchi, and pulmonary vessels. Acute pulmonary complications of leukemia include pulmonary leukostasis and leukemic clotting, leukemic cell lysis syndrome, and hyperleukocytic reaction. While some hematologic malignancies of adulthood rarely lead to alveolar proteinosis and amyloidosis, these conditions are not described in children [2,3]. Pleural involvement in leukemia/lymphoma is expected due to the primary disease or a parapneumonic effusion with chronic pleuritis sequelae as a consequence. Mediastinal lymphadenopathy or endobronchial lymphomas can lead to bronchial compression (extra- and intraluminal, respectively) with consequent atelectasis, middle lobe syndrome, and bronchial anatomic alterations [4]. 

Immune defects, primarily related to the underlying disease or being drug-induced, may facilitate infective pneumonia from typical and opportunistic viral, bacterial, and fungal pathogens. Lung infections, and the risk of possible respiratory failure and acute morbidity/mortality, can result in anatomical damages such as bronchiectasis, cavitations, and pleural adherences.

Pre-transplantation structural damage of the lung parenchyma, pleura, thoracic lymph nodes, and pulmonary vasculature may widely conditionate the post-HSCT outcome.

Immunodeficiencies are a relatively frequent indication for HSCT in the pediatric population. Such patients before HSCT often experienced severe or chronic/persistent infections, leading to chronic pulmonary consolidation, atelectasis, dystelectasis, and bronchiectasis. These post-infective structural alterations are prone to infections or chronic bacterial colonization despite the resolution of the immunodeficiency with the HSCT [5]. In addition to the infective risk linked to anatomical problems, the persistence of bacterial colonization leads to higher exposure to pathogen-associated molecular patterns (PAMPs), even if the colonization persists only into the trachea or the upper airways. This aspect is remarkable for upper airway colonization as well as for lung transplanted patients, especially those transplanted due to cystic fibrosis [6]. Chronic PAMP exposure increases the risk of developing graft versus host disease (GvHD) and its progression [7,8].

## 3. Treatment-Related Lung Toxicities

Leukemia treatments and ablation therapies increase the risk of developing pulmonary fibrosis and pleuroparenchymal fibroelastosis. Among the drugs administered in this setting, Busulfan, total body irradiation (TBI), and Bleomycin are particularly relevant. Both X-ray exposure and Bleomycin administration are known to induce pulmonary fibrosis [9,10] and are even the most common procedures to obtain mouse models of pulmonary fibrosis [11,12]. Busulfan may produce lung injury in both the acute and long term. Almost 4% of patients develop acute interstitial pneumonitis within the first 100 days from transplantation, but alveolar hemorrhage, pleural effusions, bronchospasm, and veno-occlusive disease related to Busulfan may occur [13]. On the other hand, some patients develop chronic pulmonary interstitial fibrosis with bronchiolitis obliterans, which usually becomes clinically evident after a median of 3.5 years from exposure. It is not clear whether these two entities share the exact physiopathological mechanism and which factors determine the development of acute and chronic toxicity [13]. Overall, symptoms are extraordinarily non-specific, and patients with interstitial involvement have reduced exercise tolerance, while a cough develops only at advanced stages of the disease. Therefore, drug-induced toxicity is primarily indistinguishable from pulmonary chronic GvHD (cGvHD) or bronchiolitis obliterans syndrome (BOS). Moreover, lung function tests and imaging rarely allow discrimination between these conditions, and the diagnosis relies on excluding other causes and, in particular, infective ones, such as pneumocystis infection [14]. Nevertheless, the correct diagnosis of these conditions plays a pivotal role in their management and treatment.

In children receiving HSCT, pleuroparenchymal fibroelastosis is a rare complication that affects 4% of them but carries a 73% risk of death. Its precise etiology is still a matter of debate, but it seems reasonable to point out the conditioning regimen as the most important causative factor; thus, fibroelastosis is equally distributed between recipients of allogenic and autologous HSCT, and therefore, an immunological-driven phenomenon seems unlikely [15].

## 4. Infections

The risk of systemic infections significantly represents the first cause of death in the early phases of HSCT (conditioning therapy and before engraftment). It may also persist and should always be addressed since immunosuppressive regimens may be used as GvHD prophylaxis and treatment.

Infections are closely linked to cGvHD. In the first six months after HSCT, about 28% of patients with cGVHD experience three or more infections [16]. Every infectious event may contribute to the progression of cGvHD in terms of the need to taper immunosuppressive agents but also due to immunogenic stimuli.

Bacterial pneumonia affects up to 15% of HSCT recipients. Regarding the late post-transplantation phase, encapsulated organisms, such as S. pneumoniae and H. influenzae, are frequently isolated, especially in patients with pulmonary cGvHD; tubercular and non-tubercular mycobacterial infections are rarely seen in patients with poor immune reconstitution and cGvHD [17]. Fungal infections can affect all HSCT phases, with aspergillosis as the most common invasive mold infection, followed by zygomycosis and, more rarely, mucormycosis and candida infections. Pneumocystis jiroveci pneumonia occurs less frequently due to the broadly diffused use of prophylaxis. Similarly, anti-viral prophylactic therapies reduced the burden of pneumonia caused by the reactivation of pathogens, such as herpes simplex virus-1 and -2, cytomegalovirus, human herpes virus-6 and varicella-zoster virus. However, community-acquired respiratory viruses, such as respiratory syncytial virus (RSV), influenza A and B, parainfluenza, and rhinovirus, continue to represent a significant cause of respiratory failure after transplants, with a mortality rate of up to 30% [18].

## 5. Acquired Allergies

The onset of atopy and asthma following HSCT has been reported in the literature [19] and can represent a rare complication of HSCT. An HSCT from an atopic donor to a nonatopic recipient may lead to the acquisition of mature B- and T-memory cells already sensitized to specific allergens. Memory cells may produce donor allergen-specific immunoglobulins E (IgE). In most cases, there is a spontaneous resolution within the first year after HSCT. This phenomenon may produce allergic sensitization and clinically significant allergic disease [20]. Diagnosing newly onset asthma after HSCT is challenging, but it should always be suspected in the case of symptoms. Periodic repetition of spirometry is the cornerstone for diagnosing asthma, and a bronchodilator reversibility test with albuterol should always be performed to differentiate asthma from BOS [14].

## 6. Pathogenesis of Pulmonary Chronic Graft versus Host Disease (GvHD)

The pathogenesis of cGvHD reflects a complex process involving immune dysregulation after HSCT, where the interaction between innate and adaptive immunity plays a central role. The underlying disease and conditioning regimens lead to host tissue injuries by releasing signaling molecules such as damage-associated molecular patterns (DAMPs) and consequent host innate system-driven inflammation, enhancing tissue damage and antigen release. Such a phenomenon occurs before HSCT but persists afterward. Consequently, the release of tissue-specific DAMPs, such as high mobility group box 1 (HMGB-1), adenosine triphosphate (ATP), uric acid, heat shock proteins (HSPs), and heparan sulfate proteoglycans, leads to the activation of both host-resident antigen-presenting cells (APCs) and donor-recently migrated APCs. This activation results in the presentation of alloantigens to donor T-cells with the release of pro-inflammatory cytokines, including IL-1β, IL-6, tumor necrosis factor (TNF)-α, and T-cell-stimulating cytokines such as IL-12. This escalation in the inflammatory response causes a loss in peripheral tolerance and the onset of GvHD [21]. Aβ T lymphocytes have been established as a specific population subset of cells that are deeply involved in the development of GvHD, particularly in the early phases. Therefore, a cell manipulation technique has been designed to drastically reduce the amount of αβ T lymphocytes delivered through HSCT (called αβ T-cells depleted HSCT), resulting in a significative reduction in GvHD [22]. In recent years, knowledge of the precocious phases of GvHD development has expanded greatly. The recipient’s innate lymphoid cells (ILCs) are crucial in maintaining peripheral tolerance and GvHD prevention. In particular, the ILC3 can be compared to a T-helper among ILCs, and a specific subset of the ILC3, the IL-22-producing RORγt+, plays a crucial role in maintaining epithelial cell integrity and preventing intestinal GvHD [23]. We can only infer a similar role in the precocious phase of pulmonary GvHD.

**Figure 1:**
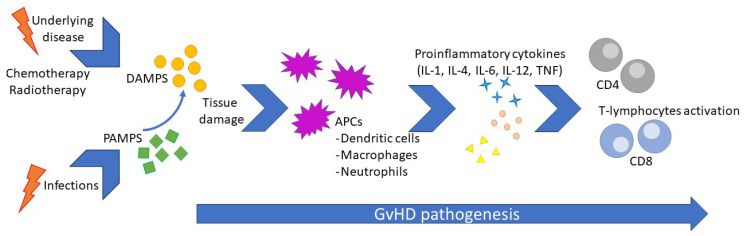
Central aspects of GvHD pathogenesis.

Infections can trigger inflammation, representing a further risk of GvHD activation or flares. During infections, pathogen-associated molecular patterns (PAMPs), such as lipopolysaccharides (LPS) and β D-glucanes, stimulate innate immunity activation, leading to tissue damage and, therefore, the release of DAMPs. This mechanism activates adaptive immunity by activating donor-derived T-cells via APC signaling [24]. Unfortunately, immunosuppressive therapy used to prevent GvHD may reduce the pathogen’s clearance and lead to prolonged or even chronic exposure to PAMPs, thus creating a vicious circle. Viruses, due to their particular intracellular damage mechanism and the consequent release of both PAMPs and DAMPs, have been pointed out as the most likely trigger for GvHD since the late eighties, and their role was confirmed after that [25,26]. It is noteworthy that a diagnosis of GvHD carries a 60% increase in the risk of infections, leading to a vicious circle due to PAMPs, and DAMP exposure leads to GvHD flares, which may require a step-up in immunosuppressive treatment [27]. Airways are the site most commonly involved in infections in the pediatric population. The central aspect of GvHD pathogenesis is resumed in Figure 1.

## 7. Pulmonary Chronic GvHD Clinical Aspects

Symptoms of cGvHD are highly non-specific and become clinically evident only at advanced stages. Therefore, close follow-up and lung function test monitoring are strongly recommended for precocious recognition of this complication. Symptoms of pulmonary cGvHD are characterized by exertional dyspnea, cough (mostly non-productive), and wheezing. In advanced phases of the disease, patients often report chest stiffness sensations with difficulty or impossibility with taking a deep breath [28]. Since GvHD results from the interaction between the donor’s immune system and the recipient tissues and the resident’s immune system, it occurs almost exclusively in allogenic HSCT from matched unrelated donors, and the frequency is much lower with haploidentical HSCT. GvHD is exceptionally rare in autologous HSCT, and these phenomena might even be a slightly different condition [29]. The baseline risk factors for cGvHD are poorly human leukocyte antigen (HLA)-matched donors, sex mismatch for male recipients [30], previous acute GvHD [31] or alveolar hemorrhage for pGvHD, the need for colony-stimulating factor to boost stem cell production, and advanced age of the donor or recipient [32]. Most of the treatments for post-transplant leukemia relapse are associated with cGvHD risk. Donor lymphocyte infusion (DLI) consist of the administration of hematopoietic stem cells of the donor into the recipient’s peripheral bloodstream to enhance the graft versus leukemia activity. DLI carries a risk of GvHD, which ranges from approximately 40 to 60% for the chronic form [33,34,35]. Applying αβ T-cell depletion to stem cells before DLI minimizes the risk of GvHD related to the procedure [35]. Immunotherapy with checkpoint inhibitors in recipients of HSCT is an established risk factor for GvHD relapse or exacerbation [36].

The historical cohort of patients with chronic pulmonary GvHD reports a 5-year survival rate of 13% [37]. Despite recent progress in GvHD management, a diagnosis of pulmonary cGvHD still carries a remarkably poor prognosis, with a 10-year survival rate ranging around 60% [38], making it the most likely cause of death after the first 4 years from HSCT [28].

## 8. Diagnosis: Lung Function Test

Chronic pulmonary GvHD is functionally identifiable by its effect on the bronchi, which is clinically defined as bronchiolitis obliterans syndrome (BOS). BOS diagnosis, according to the National Institute of Health (NIH) consensus report criteria [39], requires forced expiratory volume in the first second/forced vital capacity (FEV1/FVC) < 0.7, FEV1 < 75% of predicted, or ≥10% decline over less than 2 years, absence of respiratory tract infection, and at least one of the following supporting features of BOS: evidence of air trapping or slight airway thickening or bronchiectasis by chest computed tomography (CT), or residual volume (RV) > 120% of predicted RV divided by the total lung capacity (TLC) RV/TLC > 90%. Thus, routine spirometry should be considered mandatory in the follow-up of HSCT patients. Otherwise, often BOS following HSCT is clinically identified at late stages, when lung impairment is clinically manifest and thus severe and irreversible. In our opinion, this delay is attributable not only to inadequate lung function follow-up after HSCT (in some centers, spirometry tests are not routinely executed) but also to the suboptimal sensitivity of the spirometric parameters currently used to detect BOS (FEV1, FVC); thus, this condition can be identified late, even if the periodic follow-up is adequately performed [40].

In interpreting symptoms and spirometry, we highlight that exercise intolerance is hardly distinguishable from the expected deconditioning-related symptoms experienced during recovery after HSCT. Prolonged physical inactivity due to hospitalization and chemotherapies implies an inevitable cardio-pulmonary deconditioning due to bed rest, leading to a loss of aerobic threshold and muscle weakness [41]. This aspect, although seemingly negligible, represents the most crucial confounding variable that makes it difficult to compare pre- and post-HSCT spirometry and other pulmonary function tests. Several studies suggest that the decline in the spirometry parameters, such as the FEV1 and the forced expiratory flow between 25 and 75% of the maximum (FEF25-75), in the early post-HSCT period is a good predictor for the development of BOS [42]. Mainly, FEF25-75 seems to be a better and earlier positive predictive value than FEV1 as it measures the mid-expiratory flow volume and represents the function of terminal bronchioles, which are typically initially involved in BOS. In a recent prospective study involving 2941 allo-HCT recipients, of whom 186 developed BOS (meeting the NIH criteria), pre-transplant FEF25–75, day +80 FEV1, and day +80 FEF25–75 were statistically correlated with an increased risk of BOS. Notably, FEF25-75 had the strongest association in predicting the development of this condition [42]. Therefore, routine spirometry in patients after HSCT transplant, with the examination of FEV1 and primarily FEF25–75, plays an essential role in identifying patients with a high risk for developing BOS. We think FEF25-75 should be introduced among the diagnosis and staging criteria parameters. These individuals may benefit from pre-emptive or prompt therapy before a significant lung impairment. 

## 9. Other Functional Tests

Recent evidence suggests that oscillatory tests represent valuable tools for the early diagnosis of BOS following HSCT; even studies comparing sensitivity over spirometry are still lacking [43]. The forced oscillatory technique (FOT) is a non-invasive method that assesses pressure and flow changes in the airways by emitting oscillatory signals into the respiratory tract during tidal breathing through a mouthpiece. It could represent a valid alternative in assessing and monitoring patients who cannot perform forced expiratory blows at the spirometry test due to physical inability or a lack of compliance.

The lung clearance index (LCI), derived from multiple breath washout (MBW) testing, is another pulmonary function marker that measures ventilation heterogeneity, reflecting abnormalities of the smaller airways. Similarly to the oscillatory test, it does not require forced expiratory maneuvers, and it is currently utilized in patients affected by cystic fibrosis due to its sensibility to early airway disease. As demonstrated in several cross-sectional studies involving post-HSCT patients, LCI may have a role in the early identification of BOS since ventilation heterogeneity appeared to be an early feature of bronchiolitis obliterans [44]. Moreover, this marker seems to be more sensitive than spirometry in detecting small airway disease [45] and more feasible in young patients with scarce compliance (who are too young for conventional pulmonary function tests).

## 10. Pulmonary Chronic GvHD Treatments

Corticosteroids are considered the cornerstone of GvHD treatment. The drug of choice is methylprednisolone (1 mg/kg of up to 100 mg/day) with a slow dose tapering. Alternatively, methylprednisolone pulses (10 mg/kg) for three consecutive days given monthly for four consecutive cycles represent a valid alternative with fewer side effects than a daily regimen. However, corticosteroids have systemic side effects that limit their use, and a complete response with corticosteroids as a standalone therapy was reported in only 20% of cases, even in association with calcineurin inhibitors [39]. Therefore, steroid-sparing agents have been investigated in the last few years.

The TNF-α soluble receptor (etanercept) was tested in a phase II trial, with a good response in one-third of patients with pulmonary subacute GvHD [46]. Several other therapeutic approaches were effective in this setting, but it is not easy to establish which one should be preferred since the available evidence is mainly derived from single case reports.

Janus kinase inhibitors (JAKi), like baricitinib and ruxolitinib, are used for patients with GvHD after HSCT for leukemia. These drugs do not affect grafting; therefore, thanks to their broad availability, JAKi are now increasingly used in all forms of cGvHD [47].

Imatinib, a tyrosine kinase inhibitor that acts on the platelet-derived growth factor receptor pathway, is highly valuable for patients with refractory pGvHD [48]. JAKi and imatinib can be administered together for the treatment of pulmonary cGvHD.

Mammalian targets of rapamycin inhibitors (mTORi), such as everolimus or sirolimus, and calcineurin inhibitors were beneficial alone or in combination with mofetil mycophenolate in patients with bronchiolitis obliterans syndrome after lung transplanted patients. Consensus conferences, therefore, recommended these drugs for managing pulmonary cGvHD without a substantial body of evidence. Total lung irradiation is considered for rapidly evolutive GvHD to shut down activated lymphocytes [39].

Extracorporeal photopheresis improved one patient’s lung function and skin involvement [49], but it is an invasive technique. Mesenchymal stem cell transplantation has been described in some patients with variable cGvHD [49].

## 11. Pulmonary Fibrosis

The persistence of such a high mortality rate is mostly an indirect effect mediated by fibrosis deposition rather than a direct effect of GvHD-related inflammation.

Therefore, pulmonary GvHD therapy should aim to reduce pulmonary inflammation and directly prevent fibrosis.

Only three case reports have been published about the use of antifibrotics in HSCT-related BOS and HSCT pulmonary fibrosis [50,51,52]. Pulmonary fibrosis, although usually less rapidly evolutive, is a chronic and irreversible process. Drugs are generally intended to slow the process rather than reverse it, which seems unlikely due to its pathogenesis [52]. Thus, contrary to immunosuppressive medications, the evidence for the use of antifibrotic agents in such patients is anecdotal at its best. The use of all the antifibrotic agents should be considered off-label in the pediatric population, both for indications and age.

Despite pirfenidone being proven as effective in adults with idiopathic pulmonary fibrosis (IPF), no information is reported about its use in children, except for case reports [53,54].

Nintedanib, a tyrosine kinase inhibitor targeting the vascular endothelial growth factor receptor, the fibroblast growth factor receptor, and the platelet-derived growth factor receptor, is the only antifibrotic drug that has been studied in the pediatric population. In the PedILD trial, nintedanib resulted in an improvement in lung function tests. Despite a high prevalence of adverse effects involving 38% of children (mainly gastrointestinal), these led to drug discontinuation in only 8% of treated patients [55].

Pamrevlumab is the first monoclonal antifibrotic antibody. It is a fully human antibody of the IgG4 class directed against the connective tissue growth factor (CTGF), which is the main final effector of the tissue growth factor beta (TGF-β), profoundly inhibiting that profibrotic pathway. Pamrevlumab, a monoclonal antibody, is much more specific than pirfenidone and nintedanib, and it presents fewer side effects but needs monthly intravenous administration. Although not approved as a drug yet, pamrevlumab has already been tolerated by pediatric patients in a phase II trial conducted on Duchenne patients, showing efficacy in reducing pulmonary FVC reduction [56].

A new promising drug is BI 1015550, a preferential phosphodiesterase 4B inhibitor, which was administered via nebulization and tested to be effective for improving the FVC of adults with IPF in a phase II trial [57].

## 12. Lung Transplantation for LONIPCs

As we described, LONIPCs represent a serious complication after HSCT, being refractory to most therapeutic attempts. As for other advanced lung diseases, lung transplantation often represents the only viable option for both pediatric and adult patients with severe respiratory impairment due to LONIPCs.

The first report of lung transplantation for this condition dates back to 1992, but the literature about this option is scarce. A recent study [58] revealed an increased risk of post-lung transplantation mortality in recipients with previous allo-HSCT associated with infections; the authors hypothesized that these patients are relatively more immunosuppressed than those undergoing lung transplantation for other indications. Moreover, the sequalae of long-term steroid treatment, microbial colonization, and the risk of primary malignancy recurrence further impact their survival after transplantation.

The current international guidelines [59] establish that there must be a time interval of more than 5 years between HSCT and LT for patients with a history of hematologic malignancy.

However, a study by M. Noguchi et al. [60] found no significant difference in survival between patients with a time interval of more than 5 years and less than 5 years, suggesting that carefully selected patients with LONIPCs may be accepted as lung transplant candidates even when the time interval between HSCT and LT is less than 5 years.

Furthermore, over the last decade, there have been more publications [61,62,63] demonstrating that lung transplantation outcomes are comparable to other end-stage diseases, concluding that LT should be considered feasible in selected candidates.

## 13. Newly Discussed Parallelisms between GvHD and Pulmonary Transplant Lung Dysfunction

The multifactorial immunopathology of GvHD after HSCT is similar to the pathophysiological mechanisms responsible for developing chronic lung allograft dysfunction (CLAD) after lung transplants. Both disorders are caused by self- and non-self-discordance between donor and recipient major/minor histocompatibility antigens (donor hematopoietic stem cells against the host’s lungs in GvHD and host immune cells versus the donor’s lungs in CLAD). This condition leads to structural damage of the pulmonary parenchyma and small airways, with subsequent fibroproliferative repair processes that result in lung fibrotic remodeling. Additionally, several other mechanisms, including tissue injury from infections, radiotherapy, and conditioning regimens, contribute to the progression of these disorders by triggering an immune response. Moreover, impaired T-cell immune regulation due to thymus dysfunction or the immunosuppressive regimen with inadequate control of B-cell expansion provides further immune activation in the transplant recipient, contributing to structural damage of the lung parenchyma and small airways. All these events lead to a final common clinical syndrome called GvHD (in HSCT transplants) or CLAD (in lung transplants) [1]. GvHD, like CLAD, encompasses several pulmonary disease entities with distinct pathophysiological mechanisms, resulting in obstructive rather than restrictive phenotypes and clinical manifestations. Bronchiolitis obliterans syndrome (BOS), which affects more than 5% of HSCT recipients, is characterized by progressive and irreversible obstruction of the small airways due to bronchiolar fibrosis [64]. This syndrome is the most known and marked entity of GvHD; however, it is often recognized too late, as symptoms are mostly non-specific (cough, dyspnea on exertion or at rest) and tend to appear in the advanced stage. Furthermore, the alterations in spirometry parameters that are currently used to identify BOS onset (FEV1, FVC) are not highly sensitive as they reflect the involvement of proximal bronchi, failing to detect early flow decline in the small airways (bronchioles).

This extremely recent parallelism with CLAD revealed that pulmonary cGvHD can lead to manifestations other than BOS. Among these manifestations, we can find organizing pneumonia, pleural/pulmonary vascular complications, and interstitial lung diseases, including restrictive pulmonary cGvHD [14]. The latter clinical entity does not yet have definition criteria; however, its pathophysiologic mechanisms, radiological lung features (ground glass opacities, consolidations, linear, and reticular interstitial changes), and clinical manifestations are comparable to the well-described restrictive allograft syndrome (RAS) following lung transplantation. RAS is defined as a decline in TLC ≥ 10% when compared with the mean of the two best post-operative values, in the context of a decrease in FEV1 of ≥20%, with persistent fibrotic opacities on chest CT without obstruction [65]. Considering the significant incidence of restrictive pulmonary cGvHD after HSCT, representing 12–60% of LONIPCs [1], this clinical entity should be better categorized to improve early recognition and standardized management.

## 14. Conclusions

HSCT carries a significant risk of pulmonary dysfunction, which is often sustained by several underlying mechanisms that, in all cases, interact with each other and with the treatments needed for each of these conditions, although the clinical picture is often dominated by chronic pGvHD. It should always keep in mind the complexity of such interactions. In our opinion, the most crucial breakthrough is the recently acquired viewpoint that considers the experience of pulmonary transplanters with lung rejection and its profound similarity with pGvHD. The evidence in this field is overall scarce and outdated due to the rapid advances in immunosuppression that are already part of common practice despite the lack of adequate evidence. The literature is even more pitiful for children with pulmonary involvement related to HSCT.

## Data Availability

Not applicable.
